# Temporomandibular joint arthritis in patients with non-JIA childhood arthritis

**DOI:** 10.1186/1546-0096-10-S1-A76

**Published:** 2012-07-13

**Authors:** Randy Q Cron, Emily Fain, George Atkinson, Peter Weiser, Timothy Beukelman

**Affiliations:** 1 University of Alabama at Birmingham, Birmingham, AL, USA

## Purpose

Arthritis of the temporomandibular joint (TMJ) is a well recognized entity in patients with juvenile idiopathic arthritis (JIA). Recently, magnetic resonance imaging (MRI) evaluations have proven to have much greater sensitivity to detect arthritis compared to ultrasound and place the prevalence of TMJ arthritis in this population between 63% and 75%. There are a number of other rheumatologic diseases which can also present with TMJ inflammation [sarcoidosis, Sjögren disease, mixed connective tissue disease (MCTD)] but the prevalence of TMJ arthritis in these populations is less known. The goal of the current study is to report on TMJ arthritis in non-JIA patients with various chronic childhood arthritides.

## Methods

Between September 2007 to December 2010, 75 patients with various non-JIA arthritis associated rheumatic diseases (34 with Sjögren disease, 19 with sarcoidosis, 22 with MCTD) were identified via electronic medical records and studied. Diagnoses were based on the expert opinion of one of the 3 pediatric rheumatologist authors. TMJ arthritis was defined as the presence of fluid, synovial enhancement, or evidence of bony changes as examined by contrast enhanced TMJ MRI. Electronic medical records, including clinical progress notes, lab results, and TMJ imaging reports of the seventy-five patients were thoroughly examined and results documented.

## Results

Eighteen total non-JIA patients (25%) received MRIs of the TMJ during the study period, and 89% of those had documented arthritis elsewhere. Of the 18 MRI evaluations, 7 (39%) showed evidence of TMJ arthritis. Ten of 34 Sjögren disease patients were screened for TMJ arthritis by MRI (29%); 4/10 had positive MRI evidence of TMJ arthritis (including condylar flattening). Five of 19 sarcoidosis patients were screened by MRI (26%); 3/5 had positive MRI evidence of TMJ arthritis. Three of 22 MCTD pts were screened by MRI (13.6%); 0/3 had positive MRI evidence of TMJ arthritis. Subjective symptoms were present in 6/18 (33%), and 13/18 (72%) had clinical signs of TMJ dysfunction. Common subjective symptoms noted were pain, clicking, and locking of the jaw. Common clinical findings included jaw deviation during opening and reduced maximal interincisor distance. Only 2/7 (29%) of patients found to have TMJ arthritis had subjective symptoms. The remaining 5/7 (71%) had a positive clinical finding (jaw deviation). None of the patients had both subjective symptoms and positive clinical findings. Concurrent peripheral arthritis was present in 6/7 (86%).

## Conclusion

TMJ arthritis is present in a subset of patients with non-JIA childhood arthritis. Moreover, the TMJ arthritis in these children can be similarly destructive. Thus, a maxillofacial screening exam should be considered at each visit of a patient with non-JIA childhood arthritis, and subjective symptoms or clinical findings should prompt physicians to consider a TMJ MRI for further evaluation. We are currently evaluating the potential benefit of intra-articular triamcinolone hexactenoide injections of the TMJ in this cohort of non-JIA chronic arthritis patients.

## Disclosure

Randy Q. Cron: None; Emily Fain: None; George Atkinson: None; Peter Weiser: None; Timothy Beukelman: None.

